# The Effect of Pericellular Oxygen Levels on Proteomic Profile and Lipogenesis in 3T3-L1 Differentiated Preadipocytes Cultured on Gas-Permeable Cultureware

**DOI:** 10.1371/journal.pone.0152382

**Published:** 2016-03-29

**Authors:** Martin Weiszenstein, Nela Pavlikova, Moustafa Elkalaf, Petr Halada, Ondrej Seda, Jan Trnka, Jan Kovar, Jan Polak

**Affiliations:** 1 Center for Research on Diabetes, Metabolism and Nutrition, Third Faculty of Medicine, Charles University in Prague, Prague, Czech Republic; 2 Department of Biochemistry, Cell and Molecular Biology—Division of Cell and Molecular Biology, Third Faculty of Medicine, Charles University in Prague, Prague, Czech Republic; 3 Laboratory for Metabolism and Bioenergetics, Third Faculty of Medicine, Charles University in Prague, Prague, Czech Republic; 4 Laboratory of Molecular Structure Characterization, Institute of Microbiology of the CAS, v.v.i., Prague, Czech Republic; 5 Institute of Biology and Medical Genetics, First Faculty of Medicine, Charles University in Prague, Prague, Czech Republic; 6 Centre of Toxicology and Health Safety, The National Institute of Public Health, Prague, Czech Republic; 7 2^nd^ Internal Medicine Department, University Hospital Kralovske Vinohrady, Prague, Czech Republic; University of Minnesota—Twin Cities, UNITED STATES

## Abstract

Pericellular oxygen concentration represents an important factor in the regulation of cell functions, including cell differentiation, growth and mitochondrial energy metabolism. Hypoxia in adipose tissue has been associated with altered adipokine secretion profile and suggested as a possible factor in the development of type 2 diabetes. *In vitro* experiments provide an indispensable tool in metabolic research, however, physical laws of gas diffusion make prolonged exposure of adherent cells to desired pericellular O_2_ concentrations questionable. The aim of this study was to investigate the direct effect of various O_2_ levels (1%, 4% and 20% O_2_) on the proteomic profile and triglyceride accumulation in 3T3-L1 differentiated preadipocytes using gas-permeable cultureware. Following differentiation of cells under desired pericellular O_2_ concentrations, cell lysates were subjected to two-dimensional gel electrophoresis and protein visualization using Coomassie blue staining. Spots showing differential expression under hypoxia were analyzed using matrix-assisted laser desorption/ionization mass spectrometry. All identified proteins were subjected to pathway analysis. We observed that protein expression of 26 spots was reproducibly affected by 4% and 1% O_2_ (17 upregulated and 9 downregulated). Pathway analysis showed that mitochondrial energy metabolism and triglyceride synthesis were significantly upregulated by hypoxia. In conclusion, this study demonstrated the direct effects of pericellular O_2_ levels on adipocyte energy metabolism and triglyceride synthesis, probably mediated through the reversed tricarboxylic acid cycle flux.

## Introduction

Cultured 3T3-L1 cells are widely used as a model in adipocyte research due to their ability to differentiate and accumulate triglycerides in lipid droplets, and due to their structural and metabolic characteristics that mimick primary adipocytes [1]. Excessive intracellular lipid accumulation represents a hallmark of obesity, which is causally linked to the development of Type 2 diabetes, cardiovascular diseases and excessive mortality [2–5]. Even though *in vitro* experiments represent an indispensable tool in metabolic research, several factors, such as culture media composition, cell-to-cell interactions and cultureware surface, profoundly modify the function and structure of cultured cells (including preadipocytes), raising important concerns for interpretation and extrapolation of results. Surprisingly, little attention has been devoted to pericellular O_2_ levels, despite its documented importance in numerous cell processes, including growth, viability, differentiation and substrate metabolism [6–9].

Direct measurements as well as mathematical models have shown that pericellular O_2_ concentration differs dramatically from the predicted levels based on the air/liquid equilibrium between culture media and the surrounding atmosphere, e.g. inside the CO_2_ incubator [10]. In fact, several cell lines become severely hypoxic or anoxic when incubated under standard conditions (20% O_2_) [10,11]. Physical laws of gas diffusion in liquids, defined by diffusion distance (media volume) and molecular diffusivity, determine the amount of O_2_ transported from the gas phase above the culture media to oxygen consuming cells grown at the bottom of cultureware wells [10–12]. Pericellular oxygen levels are strongly affected by cell metabolic activity and cell number (confluency), both of which continuously change during a typical culture and/or experimental procedures. These changes in oxygen consumption rate modify the pericellular O_2_ levels, and therefore reaching and maintaining a stable pericellular O_2_ concentration represents a major challenge for *in vitro* models.

Exposure of differentiated pre-adipocytes cultured in standard cultureware to O_2_ levels ranging from 1% to 21% was previously used to better understand metabolic and endocrine changes in adipocyte function associated with adipose tissue hypoxia in obesity [13–17]. However, this experimental paradigm is limited by the fact that actual pericellular O_2_ concentrations remain largely unknown and difficult to control over a prolonged period of time. To address this limitation, the use of a gas-permeable cultureware with the bottom surface made of a fluorocarbon membrane enabling direct diffusion of O_2_ to the pericellular space is used [18].

The aim of this study was to elucidate the specific effects of various pericellular O_2_ concentrations on structural and functional aspects of 3T3L1 differentiated pre-adipocytes. To achieve this goal, 3T3L1 pre-adipocytes were differentiated at pericellular O_2_ levels of 1%, 4% and 20% using gas-permeable cultureware. Intracellular lipid accumulation was assessed and differentially expressed proteins were detected through 2-D electrophoresis followed by subsequent protein identification using MALDI-TOF mass spectrometry.

## Materials and Methods

### 3T3L1 cells culture and differentiation

Murine 3T3-L1 fibroblasts were obtained from Zen-Bio (Zen-Bio Inc., NC, USA). Cells were cultured on dishes with fluorocarbon film bottoms (Prod. #94.6077.410, Sarstedt AG & Co, Nümbrecht, Germany). Cells were expanded at cell passage number 11, for 4–5 days in T75 flasks (Prod. #156499 Nunc, Roskilde, Denmark) containing pre-adipocyte medium (PM1-L1, Zen-Bio Inc., NC, USA); subsequently, cells were harvested and plated at a cell density of 5000 viable cells/cm^2^ and grown to confluence in pre-adipocyte medium for 6 days. On day 6, the culture medium was changed to differentiation medium (DM2-L1, Zen-Bio Inc., NC, USA) and cells were placed in sealed plastic chambers—modular incubators (Billups-Rothenberg, CA, USA) flushed with calibration quality certified gas mixtures of 4% O_2_ + 5% CO_2_ or 1% O_2_ + 5% CO_2_ (Linde Gas a.s., Prague, Czech Republic) to achieve desired hypoxic environments inside the chambers. Oxygen levels inside modular incubators were continually monitored using a handheld oximeter (GOX 100, Greisinger GHM Messtechnik GmbH, Regenstauf, Germany). Chambers were placed into an incubator keeping the temperature at 37°C. Chambers were opened for media exchange every other day and immediately flushed with the gas mixture to achieve the desired O_2_ concentrations, as described above. A separate set of cells (control exposure) was placed in a standard CO_2_ incubator (5% CO_2_ + 20% O_2_). Starting three days after the beginning of differentiation, cells were fed every other day with adipocyte medium (AM2-L1, Zen-Bio Inc., NC, USA) according to the manufacturer´s instructions. Cells were used for analysis 14 days after induction of differentiation—at this stage, cells were referred to as "differentiated preadipocytes."

### Lipid accumulation quantification

Independent sets of cells were fixed for 30 min in 2% formaldehyde and subsequently stained for 2 hours with Oil Red O (Prod. No. O0625, Sigma Aldrich, St. Louis, MO, USA) as previously described [19,20]. Subsequently, cells were washed twice with phosphate buffered saline, accumulated Oil Red O was extracted with 500 μL isopropanol (Prod. No. I9516, Sigma Aldrich, St. Louis, MO, USA) and quantified spectrophotometrically at λ = 510 nm. Cell quantity was evaluated using a Total DNA kit (Prod. DNAQF, Sigma Aldrich, St. Louis, MO, USA).

### Cell culture lysates

Cells were lysed in 2-D Protein Extraction Buffer-V (GE Healthcare, Uppsala, Sweden) with a protease inhibitor cocktail (Complete Protease Inhibitor Cocktail, Roche, Basel, Switzerland) and Phosphatase inhibitor cocktail 2 and 3 (Sigma Aldrich, St. Louis, MO, USA). Lysis buffers, with inhibitor cocktails, were prepared according to the manufacturer's instructions. Cells with lysis buffer were cooled for 20 min on ice. Samples were then stored in liquid nitrogen until analyzed.

### Preparation of samples for 2-D electrophoresis

All samples were precipitated with trichloroacetic acid, followed by washing with acetone and dissolution of precipitated proteins in Protein Extraction Buffer-V supplemented with 2% Protease Inhibitor Mix (urea, thiourea, CHAPS) using a 2-D Clean-Up Kit (GE Healthcare, Uppsala, Sweden) following the manufacturer´s instructions. Purification was performed twice due to excess lipids in the samples. Protein concentrations were determined using 2-D Quant Kit (GE Healthcare, Uppsala, Sweden), which is compatible with both detergents and thiourea present in Protein Extraction Buffer-V.

### 2-D electrophoresis: isoelectric focusing

Isoelectric focusing was performed using an IPGphor focusing unit (GE Healthcare, Uppsala, Sweden). Samples were loaded on 7 cm long pH 4–7 Immobiline DryStrips (GE Healthcare, Uppsala, Sweden). Strips were rehydrated for at least 24 h with 125 μl of the diluted protein sample containing 125 μg of proteins, 2.5 μl of bromophenol blue solution 0.1%, 2 μl of IPG buffer pH 4–7 (GE Healthcare, Uppsala, Sweden) and 2.5 μl of 1 M dithiothreitol solution in Protein Extraction Buffer-V. Subsequently, pH 4–7 strips were focused at 20°C with a limited current of 50 μA/strip using the following conditions: gradient 0→150 V for 1 h, 150 V for 1 h, gradient 150→300 V for 1 h, 300 V for 2 h, gradient 300→1200 V for 3 h, 1200 V for 1 h, gradient 1200→3500 V for 5 h, and 3500 V for 5.5 h. Strips with pH 6–11 were focused at 20°C with a limited current of 50 μA/strip using the following conditions: gradient 0→150 V for 2 h, 150 V for 1 h, gradient 150→300 V for 1 h, 300 V for 2 h, gradient 300→1200 V for 3 h, 1200 V for 1 h, gradient 1200→3500 V for 5 h, and 3500 V for 1.5 h.

### 2-D electrophoresis: equilibration

Following isoelectric focusing, 20 min equilibration was performed in a buffer containing 2% DTT and 6 M urea, 30% glycerol, 4% SDS, 50 mM Tris (stock solution of 1.5 M Tris-HCl, pH 8.8). Subsequently, strips were allowed to equilibrate for another 20 min in a buffer containing 2.5% iodoacetamide instead of DTT. Finally, strips were sealed and placed on top of 0.5% agarose gels containing bromphenol blue.

### 2-D electrophoresis: SDS-PAGE

The second dimension was performed in a Mini-PROTEAN Tetra cell (Bio-Rad, Redmond, WA, USA) using 10% polyacrylamide gels with 4% stacking gels. Electrophoretic separation was run at a constant voltage of 50 V until the blue line reached the bottom of the gels (approximately 3 h). After separation, i.e., running the second dimension, gels were washed for 3x5 min in distilled water and stained overnight in 50 ml of colloidal Coomassie blue solution [21].

### Gel image and analysis

Stained gels were digitally imaged using a calibrated UMAX PowerLook 1120 scanner with LabScan software (both GE Healthcare, Uppsala, Sweden) and analyzed using Image Master^TM^ 2D Platinum 6.0 software (GE Healthcare, Uppsala, Sweden). Differences between corresponding spots were analyzed in each set of gels (control vs. hypoxia 1 h and hypoxia 4 h) and selected spots with twofold (or higher) average difference in expression between control and any of the hypoxia samples, were cut and sent for mass spectrometry identification.

### Enzymatic digestion

Selected spots were excised from gels, cut into small pieces and destained using 50 mM 4-ethylmorpholine acetate (pH 8.1) in 50% acetonitrile (MeCN). Subsequently, gels were washed with water, reduced in size by dehydration in MeCN and then reconstituted in water. A SpeedVac concentrator was used to partly dry the gel, while supernatant was removed. Gel pieces were then incubated overnight at 37°C in cleavage buffer containing 25 mM 4-ethylmorpholine acetate, 5% MeCN and trypsin (100 ng; Promega). Finally, extraction of resulting peptides was performed with 40% MeCN/0.1% TFA (trifluoroacetic acid).

### MALDI mass spectrometry and protein identification

The MALDI matrix consisted of an aqueous 50% MeCN/0.1% TFA solution of α-cyano-4-hydroxycinnamic acid (5 mg/ml; Sigma-Aldrich, St. Louis, MO, USA). 1 μl of the peptide mixture was deposited on a MALDI plate, allowed to air-dry, at room temperature, and overlaid with 0.4 μl of matrix. Mass spectra were measured using an Ultraflex III MALDI-TOF (BrukerDaltonics, Bremen, Germany); mass range of 700–4000 Da, calibrated internally using the monoisotopic [M+H]^+^ ions of trypsin auto-proteolytic fragments (842.5 and 2211.1 Da). The peak lists, created using flexAnalysis 3.3 software, were analyzed using an in-house MASCOT search engine against the SwissProt 2015_10 database subset of mouse proteins with the following search settings: peptide tolerance of 30 ppm, missed cleavage site value set to one, variable carbamidomethylation of cysteine, oxidation of methionine and protein N-term acetylation. Proteins with MOWSE scores over the threshold, 56 (calculated for the settings used) were considered as identified. If necessary, the identity of a protein candidate was confirmed using MS/MS analysis.

### Statistical analysis

For 2-DE gel analysis, the statistical significance of the changed expression of individual spots was determined using Student´s t-test. Pathway analysis of identified proteins was performed using Ingenuity Pathways Analysis (Qiagen, Redwood City, CA, USA). Statistical significance was set at p < 0.05.

## Results

### Lipid synthesis and cell count

**Fig 1:**
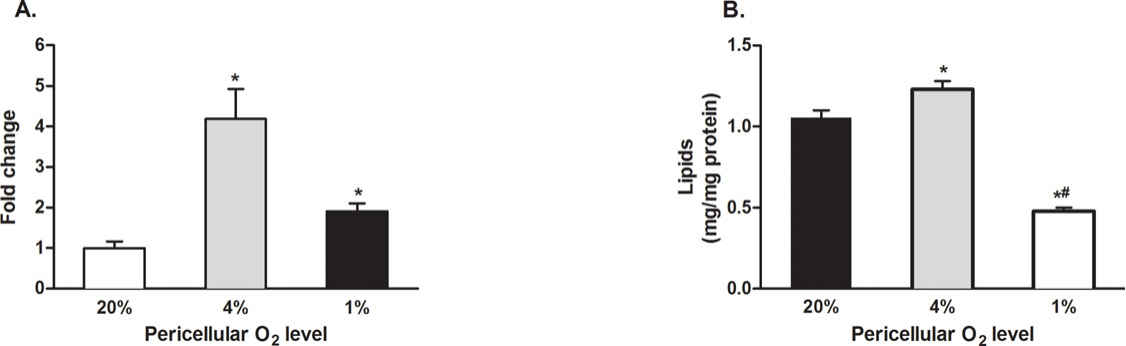
Triglyceride accumulation under exposure to 1%, 4% and 20% O_2_. 3T3-L1 cells were cultured and differentiated under desired O_2_ concentrations for 14 days. Lipid accumulation was evaluated using Oil Red O staining. (A) Data are expressed as fold-change relative to a control condition (20% O_2_), (B) Data are normalized to total protein content. * p < 0.05 for comparison with 20% O_2_.

Adipocytes cultured and differentiated under 4% O_2_ accumulated 4.2 ± 0.8 resp. 2.2 ± 0.2 times more lipids than cells exposed to 20% O_2_ or 1% O_2,_ Fig 1A. These effects were diminished, but remained statistically significant, when lipid amounts were normalized to protein content (1.0 ± 0.1, 1.2 ± 0.1 and 0.5 ± 0.1 mg lipids/mg protein for 20%, 4% and 1%, respectively, all p < 0.05), Fig 1B. Furthermore, when compared to cells cultured under 20% and 1% O_2_, growing adipocytes under 4% O_2_ improved cell viability (expressed as cell counts in wells at the end of the differentiation period) by 220 ± 20% and 230 ± 40%, respectively, (p < 0.05).

### 2-D electrophoresis and MALDI-TOF mass spectrometry

**Fig 2:**
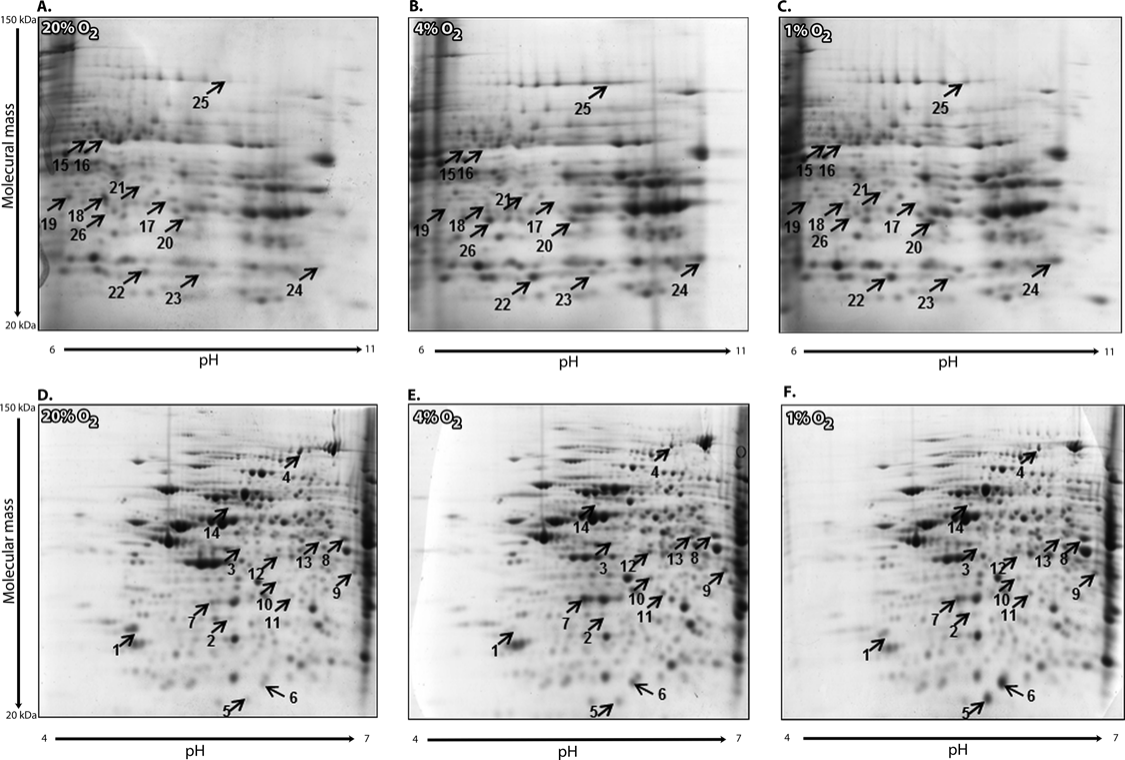
Representative examples of 2-DE gels. Representative 2-DE gels of 3T3-L1 differentiated preadipocytes exposed to 1% and 4% hypoxia compared to control exposure (20%). Gels contain visualized proteins with isoelectric point within pH range 6–11 (A, B, C) and 4–7 (D, E, F). Differentially expressed proteins are marked by arrows and numbers.

**Fig 3:**
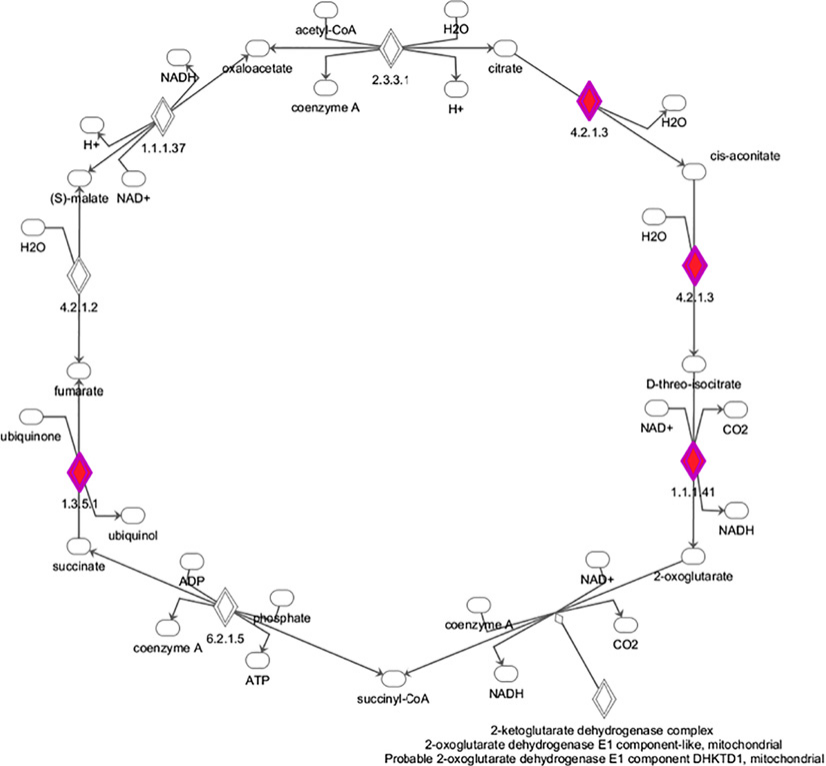
A graphical interpretation of the identified proteins in the reversed TCA cycle. Tricarboxylic acid cycle is depicted with marked proteins upregulated by exposure to 1% O_2_ and 4% O. Glutamine enters the reversed TCA cycle as α-ketoglutarate and is subsequently processed to citrate and acetyl-CoA. *Enzymes in metabolic pathways are shown as diamonds accompanied by enzyme nomenclature numbers*, *oval symbols represent substrates or products of metabolic reactions*. *Colored diamonds represent enzymes identified in proteomic analysis*. *1*.*3*.*5*.*1 –Succinate dehydrogenase*, *4*.*2*.*1*.*3 –Aconitate hydratase*, *1*.*1*.*1*.*41 –Isocitrate dehydrogenase*.*"*

Approximately 400 stable spots with isoelectric point (pI) 4–7 and 300 stable spots with pI 6–11 with a molecular weight 20–150 kDa were detected on each 2-DE gel Fig 2. The gels were matched using Image Master^TM^ software and subsequently checked manually for accuracy. Intensities of corresponding spots were analyzed for appropriate pairs of gels (20% O_2_ versus 1% O_2_ and 20% O_2_ versus 4% O_2_) and spots with a 2-fold higher or 2-fold lower intensity, were identified as differentially expressed proteins. Analyzed expression profiles of proteins with isoelectric points between a pH range of 4.0–7.0 and molecular mass of adipocytes cultured under different pericellular O_2_ levels exhibited high reproducibility, relative to individual sets of samples. In contrast, significant differences in expression profiles were observed between adipocytes exposed to hypoxic (1% O_2_ or 4% O_2_) and control conditions (20% O_2_). Fig 3

In total, 26 spots were identified as proteins exhibiting reproducibly changed expression across both hypoxic conditions and considered to be proteins regulated by pericellular O_2_ levels. Among these, 17 were upregulated and 9 were downregulated in adipocytes exposed to hypoxia, as summarized in Tables 1 and 2. To better understand the functional consequences of hypoxic exposure, proteomic datasets were subjected to pathway analysis, which revealed, that differentially expressed proteins belong to tricarboxylic acid cycle, branched chain amino acid metabolism, and processes associated with mitochondrial dysfunction Fig 2. In a subsequent analysis, we identified proteins following specific expression patterns: a) a pattern mimicking the functional metabolic parameter represented as intracellular lipid accumulation (increased under 4% O_2_ followed by a reduction under 1% O_2_) and b) a dose-response pattern (i.e. proteins more up/down-regulated under 1% O_2_ compared to 4% O_2_). According to pathway analysis, the majority of proteins predominantly upregulated under 4% O_2_ were involved in fatty acid metabolism and lipid synthesis (isovaleryl-CoA dehydrogenase, isocitrate dehydrogenase, acetyl-CoA acetyltransferase, aconitate hydratase, glycerol-3-phosphate dehydrogenase), while proteins that were downregulated more under 4% O_2_ belonged to structural and cytoskeletal cell components.

**Table 1:** Proteins upregulated in 1% O_2_ and 4% O_2_ compared to control conditions (20% O_2_).

Spot No.	20% vs 1%	20% vs 4%	Protein name	DTB No.	Location	No. peptides	[%] Coverage	MS/MS confirmation	MW	pI
5	5.4	3.4	Ferritin heavy chain	FRIH_MOUSE	C,N,M	7	58	YFLHQSHEEREHAEK	21	5.5
6	6.7	2.9	Ferritin light chain 1	FRIL1_MOUSE	C	7	38	VAGPQPAQTGAPQGSLGEYLFER	21	5.7
7	2.9	2.4	Inorganic pyrophosphatase	IPYR_MOUSE	C	9	37	No	33	5.4
8	2.1	1.9	Aldehyde dehydrogenase, mitochondrial	ALDH2_MOUSE	M	14	35	No	57	7.5
9	1.7	2.6	Isovaleryl-CoA dehydrogenase, mitochondrial	IVD_MOUSE	M	11	36	HSILPVDDDINGLNEEQKHSILPVDDDINGLNEEQKQLR	46	8.5
10	2.7	3.0	Isocitrate dehydrogenase [NAD] subunit alpha, mitochondrial	IDH3A_MOUSE	M	9	29	No	40	6.3
11	2.5	2.2	Transaldolase	TALDO_MOUSE	C,N	13	32	FAADAIKLER LIELYKEAGVGKDR AAQTSDSEKIHLDEKAFR	37	6.6
12	1.6	3.4	2-oxoisovalerate dehydrogenase subunit alpha, mitochondrial	ODBA_MOUSE	M	14	41	No	50	8.1
13	1.3	3.1	Lipoamide acyltransferase, mitochondrial	ODB2_MOUSE	M	13	30	No	53	8.8
14	2.4	0.8	Heterogeneous nuclear ribonucleoprotein K	HNRPK_MOUSE	C,N	5	14	NLPLPPPPPPR	51	5.4
20	1.9	1.7	L-lactate dehydrogenase A	LDHA_MOUSE	WC	11	43	No	36	7.6
21	2.6	5.9	Acetyl-CoA acetyltransferase, cytosolic	THIC_MOUSE	WC	6	20	ILVTLLHTLER AGHFDKEIVPVLVSSRVNIDGGAIALGHPLGASGCR	41	7.2
22	2.1	1.7	Triosephosphate isomerase	TPIS_MOUSE	N	12	50	No	32	5.6
23	2.1	2.0	Triosephosphate isomerase	TPIS_MOUSE	N	9	36	No	32	5.6
24	2.7	2.4	Succinate dehydrogenase [ubiquinone] iron-sulfur subunit, mitochondrial	SDHB_MOUSE	M	10	29	No	32	9
25	1.8	3.9	Aconitate hydratase, mitochondrial	ACON_MOUSE	M	15	26	SQFTITPGSEQIR NAVTQEFGPVPDTARWVVIGDENYGEGSSR	85	7.9
26	2.0	6.9	Glycerol-3-phosphate dehydrogenase [NAD(+)], cytoplasmic	GPDA_MOUSE	M	7	16	VCYEGQPVGEFIR	37	6.8

Table provides spot number, change in intensity between spots from cells cultured in 1% O_2_ or 4% O_2_ versus 20% O_2_, protein name, SwissProt database number, location of protein within cell [C (cytosol), N (nucleus), M (mitochondria), WC (whole cell)], number of peptides matched to the identified protein, sequence coverage, peptide sequences confirmed by MS/MS, protein molecular weight (MW) and pI values.

**Table 2:** Proteins downregulated in 1% O_2_ and 4% O_2_ compared to control conditions (20% O_2_).

Spot No.	20% vs 1%	20% vs 4%	Protein name	DTB No.	Location	No. peptides	[%] Coverage	MS/MS confirmation	MW	pI
1	0.5	0.3	Tropomyosin alpha-3 chain isoform Tpm3.1cy	359279904*	C	13	67	IQLVEEELDRAQER KIQVLQQQADDAEERAERKLVIIEGDLERTEERAELAESR	29	4.7
2	0.7	0.4	Annexin A4	ANXA4_MOUSE	C,MB	11	39	No	36	5.4
3	0.4	0.6	Cytochrome b-c1 complex subunit 1, mitochondrial	QCR1_MOUSE	M	10	26	TATFAQALQSVPETQVSILDNGLR	53	5.8
4	0.6	0.5	Vinculin	VINC_MOUSE	C,MB	17	27	No	117	5.8
15	0.4	0.5	Glutamate dehydrogenase 1, mitochondrial	DHE3_MOUSE	M	14	28	No	61	8.1
16	0.4	0.7	Glutamate dehydrogenase 1, mitochondrial	DHE3_MOUSE	M	11	23	No	61	8.1
17	0.5	0.3	Annexin A1	ANXA1_MOUSE	WC	10	34	No	39	7.0
18	0.4	0.2	Annexin A1	ANXA1_MOUSE	WC	11	39	No	39	7.0
19	0.6	0.2	Annexin A1	ANXA1_MOUSE	WC	12	41	No	39	7.0

Table provides spot number, change in intensity between spots from cells cultured in 1% O_2_ or 4% O_2_ versus 20% O_2_, protein name, SwissProt database number, location of protein within cell [C (cytosol), N (nucleus), M (mitochondria), MB (membrane), WC (whole cell)], number of peptides matched to the identified protein, sequence coverage, peptide sequences confirmed by MS/MS, protein molecular weight (MW) and pI values.

*NCBI database number is shown.

## Discussion

Using gas permeable membrane-bottom cultureware that enabled prolonged and predictable exposure of cells to desired pericellular O_2_ levels, this study demonstrated the direct effects of variable oxygen availability on the proteomic profile in 3T3-L1 differentiated preadipocytes. Using pathway analysis, we found that affected pathways participate in the regulation of energy metabolism, including numerous enzymes of the TCA cycle, glycolysis, and triglyceride synthesis. At the functional level, we confirmed suggested pathway changes by showing that hypoxia stimulated triglyceride accumulation.

Direct measurements of adipose tissue oxygen levels in obese mice and humans recorded levels as low as 2–7% [22,23] underscoring the need for tools enabling modeling of such environment. Unique properties of the membrane-bottom cultureware enabled exposure of adherent cells to the desired pericellular O_2_ levels for a period of 14 days. Our group has previously showed that pericellular O_2_ levels differ markedly when cells are grown on traditional, plastic (polystyrene) cultureware compared with membrane-bottom cultureware [18]. Limited gas diffusion and constant uptake of oxygen by adherent cells leads to constantly changing pericellular O_2_ levels and ultimately results in severely hypoxic or anoxic environments in the pericellular space [10–12], which can be prevented by using the membrane-bottom cultureware [18]. By precise dosing of hypoxic exposure of various severity, this study described previously unreported aspect of adipocyte biology by demonstrating increased triglyceride accumulation in preadipocytes exposed to mild hypoxia (4% O_2_), while no such effect was observed under severe hypoxia (1% O_2_). Such non-linear association between pericellular O_2_ levels and biological effects has important clinical extrapolations, as it has been reported, that adipose tissue becomes mildly hypoxic not only in obesity, but also during sleep in subjects with obstructive sleep apnea syndrome (OSA). Direct measurements of adipose tissue O_2_ levels in an animal model of OSA showed that during intermittent hypoxic exposure (modeling oxygen desaturations observed in subjects with OSA) adipose tissue O_2_ levels reached 4–5% [24]. Our study extends previous knowledge by demonstrating that exposure of differentiated preadipocytes to clinically relevant hypoxia (4% O_2_), but not more severe hypoxia of 1% O_2_, stimulates triglyceride accumulation and provide thus a possible mechanistic explanation for the development of obesity and associated metabolic impairments in OSA.

Investigating patterns of differentially expressed proteins, proteomic and pathway analysis subsequently identified upregulated fatty acid metabolism and lipid synthesis enzymes as possible mediators driving increased triglyceride synthesis under 4% O_2_ via accelerated formation of glycerol 3-phosphate combined with increased synthesis of acetyl-CoA in the TCA cycle. Importantly, exposure to 1% O_2_ was not associated with changes in lipogenesis pathways which demonstrates the importance of precise control of pericelular O_2_ levels during in-vitro experiments. Maintenance of mitochondrial metabolic functions is a necessary prerequisite for cell survival and proliferation under limited O_2_ availability. In normoxic conditions, tricarboxylic acid cycle supplies intermediates for mitochondrial electron transport chain and subsequent ATP production. However, important changes in TCA flux have been observed when O_2_ levels were decreased. In fact, the direction of multiple TCA reactions is reversed during mitochondrial adaptation to hypoxia, since several TCA cycle enzymes (namely isocitrate dehydrogenase, aconitase and succinate dehydrogenase) can catalyze reactions in the opposite direction than the usual TCA flux from citrate to ketoglutarate [25,26]. Through the use of reversed TCA reactions, a new mitochondrial metabolic milieu is established, where glutamine becomes the predominant source of alpha-ketoglutarate, subsequently undergoing reductive carboxylation by isocitrate dehydrogenase and conversion to citrate by aconitase [25]. Mitochondrial adaptation to hypoxia has been largely investigated in the context of malignant tumors, where O_2_ availability represents a limiting factor for tumor growth. Even though it has been shown that melanoma cells exposed to hypoxia derive most of the carbon for acetyl-CoA synthesis from glutamine rather than glucose [27], it remained unclear whether other cell types could use reverse TCA cycle as an adaptation to hypoxia. Our study documented that isocitrate dehydrogenase and aconitase (key enzymes enabling reverse TCA cycle flux) were upregulated during hypoxic exposure, providing evidence that adipocytes are able employ similar mitochondrial adaptations to hypoxia previously observed in cancer cells [28]. We further suggest, that this hypoxic adaptation and reverse TCA cycle flux is causally linked to increased triglyceride deposition reported in this study.

The impact of lowered pericellular O_2_ on adipocyte metabolic and endocrine functions has been demonstrated in studies showing that reduction of O_2_ levels by ~20–30 mmHg, typically observed in obese rodents and human subjects due to increased adipocyte size and reduced tissue capillarity [16,23,29], was associated with increased lipolysis and a modified spectrum of secreted adipokines, possibly through activation of HIF-1 pathway [30]. In line with these studies, it has been demonstrated that exposure of adipocytes to hypoxia *in vitro* modifies secretion profiles as well as gene expression and induces cellular insulin resistance. In fact, it has been shown, that activation of HIF transcriptional factor occurs under severe hypoxia with a maximal effect at 0.5–1% O_2_ [18,31]. We thus hypothesize, that lipogenesis stimulating effects of mild hypoxia (4% O_2_) might be, at least partially, driven by HIF-independent mechanisms. Our observations also suggest, that optimal pericellular O_2_ levels that maximize lipogenesis during *in vitro* experiments might be close to 4% O_2_, similar to levels observed *in vivo*. In contrast, too high (20%) as well too low (1%) O_2_ levels compromise cell metabolic pathways leading to lower triglyceride content. We suggest, that increased levels of isocitrate dehydrogenase and aconitase stimulate reverse TCA cycle flux and provide acetyl-CoA, which is subsequently utilized by ATP-dependent citrate lyase for fatty acid synthesis [28]. This notion is further supported by reports documenting that carbons originating from glutamine and undergoing reverse TCA cycle were found in newly synthesized fatty acids [32]. Interestingly, glutamine was identified as a major source of acetyl-CoA for fatty acid synthesis in brown adipocytes even under normoxic conditions [33], suggesting that reverse TCA flux is not unique to hypoxia.

Besides stimulating acetyl-CoA production, we also identified that hypoxia upregulated a key enzyme in lipid synthesis, glycerol-3-phosphate dehydrogenase. Reaction catalyzed by this enzyme provides glycerol backbone, which is subsequently esterified with fatty acids to form triglycerides. Production of glycerol phosphate from glycolysis-derived DHAP represents a rate limiting step in triglyceride synthesis, because adipocytes posses only negligible glycerol kinase activity and cannot thus utilize glycerol for TG synthesis [34,35]. Upregulation of glycerol synthesis combined with augmented de novo synthesis of FFA stimulated by reversed TCA cycle could mechanistically explain our observation of enhanced lipid accumulation in cells exposed to hypoxia. Additionally, glycerol-3-phosphate has another independent function—it feeds electrons to the electron transport chain at the outer surface of the inner mitochondrial membrane and thus increases mitochondrial ROS production on Complex III [36]. Generated superoxide participates in the stabilization of HIF protein, a master hypoxia-regulated transcriptional regulator [37], which has been shown to mediate hypoxia-induced lipogenesis [38].

Interpretation of results presented in this study should take into account its limitations. Using membrane-bottom cultureware enabled us to expose cells to defined pericellular O_2_ levels, however, culturing cells on membrane surface has been shown previously to independently modify the protein expression profile [39]. It is thus possible, that cells cultured on different surfaces could show different patterns of hypoxia-induced responses. Secondly, we have investigated differences in proteomic profiles after exposure to 1% and 4% O_2_ concentrations, which are lower O_2_ concentrations than those reported in adipose tissue of obese subjects, resembling more tissue O_2_ levels expected in states accompanied by arterial hypoxemia, such as in obstructive sleep apnea syndrome or pulmonary diseases. Additionally, hypoxic exposure, particularly to 4% O_2_ also enhanced cell number and protein levels, suggesting the affect of hypoxia on adipocyte differentiation or viability. It is thus possible, that part of the observed effects of exposure to 4% O_2_ might be mediated by increased cell number/viability and augmented differentiation. Finally, we demonstrated the direct effects of O_2_ levels on proteomic profiles in 3T3-L1 differentiated preadipocytes, but changes induced by hypoxia in the whole organism and adipose tissue are more complex, including modifications in endocrine systems, such as elevated concentrations of cortisol and catecholamines. It thus remains to be investigated, whether similar adaptations to hypoxia are present in human cultured or primary adipocytes or in adipose tissue *in vivo*. Finally, inherent limitations of any proteomic-based study is the absence of a testable hypothesis at the beginning of the study. Proteomic and pathway analyses in this study provide a preliminary scientific basis for subsequent extensive validation of suggested mechanisms in more complex systems such as specific tissues or the whole organism. Future experiments should utilize antibody-based protein identification methods to validate hypotheses arising from this study, as these methods were not employed in the present study.

In conclusion, we demonstrated that various pericellular O_2_ levels differentially regulate cellular functions in differentiated preadipocytes, particularly pathways involved in intracellular triglyceride accumulation and mitochondrial metabolism. Through precise control of pericellular O_2_ levels, this study demonstrated that exposure to mild (4% O_2_), but not severe (1% O_2_), hypoxia stimulated intracellular lipid accumulation. Proteomic and pathway suggested that likely mechanisms driving triglyceride accumulation under 4% O_2_ are increased production of glycerol-3-phosphate dehydrogenase and enzymes enhancing acetyl-CoA production in TCA cycle. Presented observations not only help to elucidate mechanisms of excessive triglyceride accumulation and obesity development in states characterized by adipose tissue hypoxia (e.g. obstructive sleep apnea syndrome), but also underscore the importance of precise control of pericellular O_2_ levels in the design and interpretation of *in vitro* experiments.

## Supporting Information

S1 File Summary of data on triglyceride accumulation. (XLSX)
